# Optimization-Incorporated Deep Learning Strategy to Automate L3 Slice Detection and Abdominal Segmentation in Computed Tomography

**DOI:** 10.3390/bioengineering12040367

**Published:** 2025-03-31

**Authors:** Seungheon Chae, Seongwon Chae, Tae Geon Kang, Sung Jin Kim, Ahnryul Choi

**Affiliations:** 1 Department of Bio-Mechatronic Engineering, College of Biotechnology and Bioengineering, Sungkyunkwan University, Suwon 16419, Republic of Korea; chd8806a@skku.edu; 2 Department of Biomedical Engineering, College of Medical Convergence, Catholic Kwandong University, Gangneung 25601, Republic of Korea; cso5149@cku.ac.kr; 3 Institute for Trauma Research, College of Medicine, Korea University, Seoul 02708, Republic of Korea; kangtg@daum.net; 4 Department of Urology, Gangneung Asan Hospital, University of Ulsan College of Medicine, Gangneung 25440, Republic of Korea; 5 Department of Biomedical Engineering, College of Medicine, Chungbuk National University, Cheongju 28644, Republic of Korea

**Keywords:** computed tomography, L3 slice detection, abdominal tissue segmentation, optimization, deep learning

## Abstract

This study introduces a deep learning-based strategy to automatically detect the L3 slice and segment abdominal tissues from computed tomography (CT) images. Accurate measurement of muscle and fat composition at the L3 level is critical as it can serve as a prognostic biomarker for cancer diagnosis and treatment. However, current manual approaches are time-consuming and prone to class imbalance, since L3 slices constitute only a small fraction of the entire CT dataset. In this study, we propose an optimization-incorporated strategy that integrates augmentation ratio and class weight adjustment as correction design variables within deep learning models. In this retrospective study, the CT dataset was privately collected from 150 prostate cancer and bladder cancer patients at the Department of Urology of Gangneung Asan Hospital. A ResNet50 classifier was used to detect the L3 slice, while standard Unet, Swin-Unet, and SegFormer models were employed to segment abdominal tissues. Bayesian optimization determines optimal augmentation ratios and class weights, mitigating the imbalanced distribution of L3 slices and abdominal tissues. Evaluation of CT data from 150 prostate and bladder cancer patients showed that the optimized models reduced the slice detection error to approximately 0.68 ± 1.26 slices and achieved a Dice coefficient of up to 0.987 ± 0.001 for abdominal tissue segmentation-improvements over the models that did not consider correction design variables. This study confirms that balancing class distribution and properly tuning model parameters enhances performance. The proposed approach may provide reliable and automated biomarkers for early cancer diagnosis and personalized treatment planning.

## 1. Introduction

Cancer remains one of the most significant global public health challenges, with its incidence continuing to rise [1,2]. Notwithstanding advances in early diagnosis and treatment, many cancers still show poor prognosis due to local recurrence or distant metastasis [3,4]. Recent studies have indicated complex relationships between major cancers and various physiological axes within the human body, revealing associations between bodily components and cancer that could potentially be leveraged for treatment [5,6]. In particular, studies highlighting correlations between prostate cancer and the gut microbiome have raised the concept of the “prostate–gut axis” [7]. Such research suggests that alterations in the gut microbiome composition can influence the development and progression of gastrointestinal cancers, from changes in immune responses and metabolic pathways that affect cancer risk and patient prognosis [8,9]. Understanding these interactions within human systems underscores the importance of cancer research, while highlighting the role of body composition as a critical factor in cancer prognosis and treatment [10,11].

Changes in body composition are believed to be closely linked to alterations in the gut microbiome, which may be a result, process, or even cause of microbial imbalance [12]. These changes thus hold promise as new biomarkers for early cancer diagnosis and prognosis management [13]. Computed tomography (CT) is considered the gold standard to quantify muscle and fat within the human body, due to its ability to provide clear anatomical insights for body composition analysis [14,15]. Notably, the distribution of body composition at the L3 vertebral level in CT slices has shown a linear correlation with the overall body composition [16,17]. However, in terms of maintaining accuracy, it is both labor-intensive and challenging to identify the L3 slice and manually segment body composition in full-body CT scans [18,19]. Therefore, simplifying and accurately measuring these components remain significant issues.

Recent advances in deep learning models have demonstrated the potential to quickly, accurately, and objectively extract L3 slices and classify body composition [18,19,20,21,22]. An algorithm to detect the L3 region by applying maximum intensity projections to the coronal and sagittal planes of entire CT scans was proposed by Belharbi et al. [19]. Additionally, a Unet deep learning model capable of automatically segmenting muscles, sub-cutaneous fat, and visceral fat in L3 slices was developed, achieving a high Dice coefficient ranging from 0.90 to 0.96 [21]. More recently, an attention-enhanced deep learning model that integrates attention gates into a U-Net backbone was developed, achieving Dice scores of 0.954 for subcutaneous adipose tissue and 0.920 for skeletal muscle, outperforming classic models [22]. However, L3 slices account for approximately 5 % of abdomen and pelvic CT images, and the fat and muscle composition within these slices represent only ~10 % of total body composition. This class imbalance in the dataset degrades the performance in learning models. To address this, attempts have been made to balance data ratios by adding L3 slices from other CT datasets [23]. However, when additional data cannot be obtained, such approaches are not feasible, leaving fundamental issues of class imbalance unresolved.

The basic premise of training models is that to ensure performance, datasets must have a balanced number of samples for each classification class [24,25]. Research has indicated that when applied to test or external datasets, imbalanced data distributions can lead to overfitting during training, resulting in a sharp drop in performance [26]. Solutions to this problem broadly fall into two classes: data-level and algorithm-level approaches [27]. At the data level, the number of samples per class can be adjusted by over- or under-sampling, while at the algorithm level, class weights can be adjusted in the output layer to prevent biased learning [28]. However, the appropriate sampling levels and class weight values are often determined empirically [29,30], resulting in uncertainty about identifying the optimal values for specific models.

The objective of this study is to propose an optimization-incorporated deep learning strategy that detects L3 slices and segments abdominal tissues from CT images. The specific objectives are as follows:

1. To address the imbalance of target classes, an optimization process is proposed in which the augmentation ratio and class weight adjustments are considered as correction design variables (CDVs), and the objective function is defined based on the performance of the training models.
2. The proposed optimization-integrated deep learning strategy is validated using various state-of-the-art deep learning techniques.
3. The proposed strategy is applied to human CT images to extract the L3 slice and segment abdominal tissues.

This study hypothesizes and aims to verify that securing optimal sampling levels and class weight values for imbalanced data can enhance the performance of deep learning models.

The remainder of this paper is organized as follows: Section 2 introduces deep learning strategies to detect L3 slices and segment abdominal body composition. Section 3 describes the datasets used for model implementation. Section 4 details data preprocessing, model configuration, and post-processing to implement the deep learning strategy. Section 5 presents performance evaluation methods for the proposed strategy, while Section 6 provides the training process and results. Finally, Section 7 interprets, discusses the limitations of, and concludes this study.

## 2. L3 Slice Detection and Abdominal Segmentation Strategy

**Figure 1:**
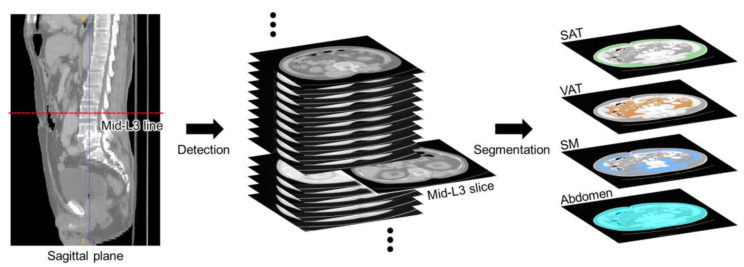
Detection of L3 slices and segmentation of abdominal tissues in abdomen and pelvic CT images.

The proposed strategy aims to identify the L3 slice from the abdomen and pelvic CT scan of each patient, and segment the abdomen, skeletal muscle (SM), subcutaneous adipose tissue (SAT), and visceral adipose tissue (VAT) within the identified L3 slice (Figure 1). This strategy employs a deep learning model for L3 slice detection and abdominal tissue segmentation (Section 2.1). It also includes an optimization process that uses augmentation ratio and class weights as CDVs to address performance degradation resulting from data imbalance (Section 2.2).

### 2.1. Deep Learning Architectures

**Figure 2:**
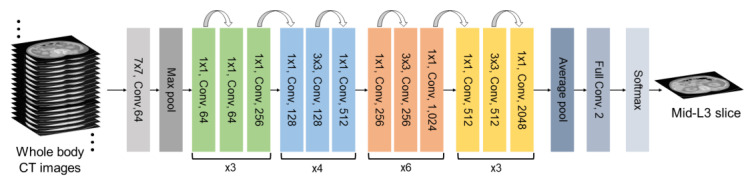
Deep learning architecture for L3 slice detection.

To detect the L3 slice in CT scans, the study employs a residual network (ResNet) 50 model based on a convolutional neural network (CNN) (Figure 2). ResNet is designed to predict the residuals required for final predictions from one layer to the next [31]. By allowing gradients to flow through alternative shortcut paths, ResNet mitigates the problem of vanishing gradients. When a particular layer is unnecessary, the identity mapping used in ResNet allows the model to bypass CNN-weighted layers, helping to avoid overfitting on the training set.

**Figure 3:**
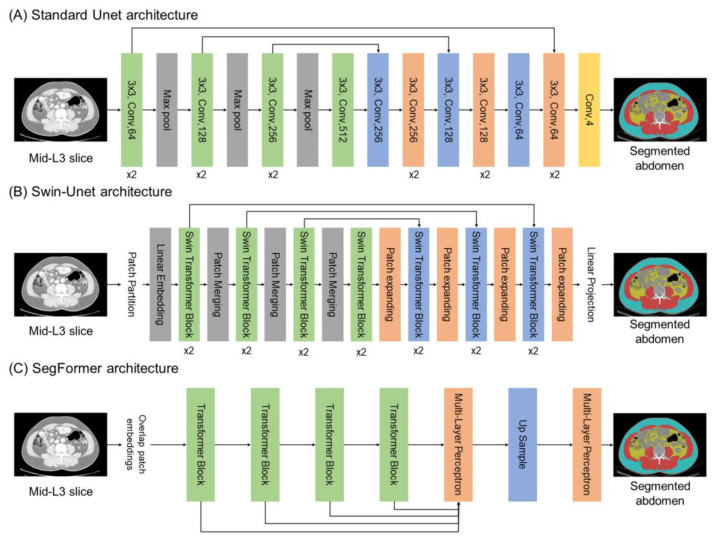
Deep learning architecture for abdominal segmentation: standard Unet (**A**), Swin-Unet (**B**), and SegFormer architectures (**C**).

Abdominal segmentation utilized three different deep learning architectures—standard Unet, Swin-Unet, and SegFormer models—each employing an encoder-decoder structure optimized for semantic segmentation tasks (Figure 3). The standard Unet model is a CNN that captures class features using a contracting encoder path and performs upsampling through an expansive decoder path (Figure 3A) [32]. It primarily employs 3 × 3 convolution operations with each convolutional block consisting of two 3 × 3 convolutions separated by a dropout layer. Max-pooling is used to reduce feature map size and skip connections between encoder and decoder layers help retain spatial information for precise localization. The Swin-Unet, inspired by the Swin Transformer, combines the strengths of the Unet architecture with the power of self-attention [33] (Figure 3B). It applies a shifted window mechanism to extract both local and global dependencies, enabling expressive feature representation. Like Unet, it maintains skip connections between corresponding encoder and decoder layers to preserve fine-grained details. The SegFormer adopts a hierarchical Transformer-based encoder that efficiently captures multi-scale contextual information without requiring positional encoding [34] (Figure 3C). Its lightweight MLP decoder aggregates features from multiple levels to produce high-resolution segmentation outputs, offering both speed and accuracy.

### 2.2. Optimization Approach

**Figure 4:**
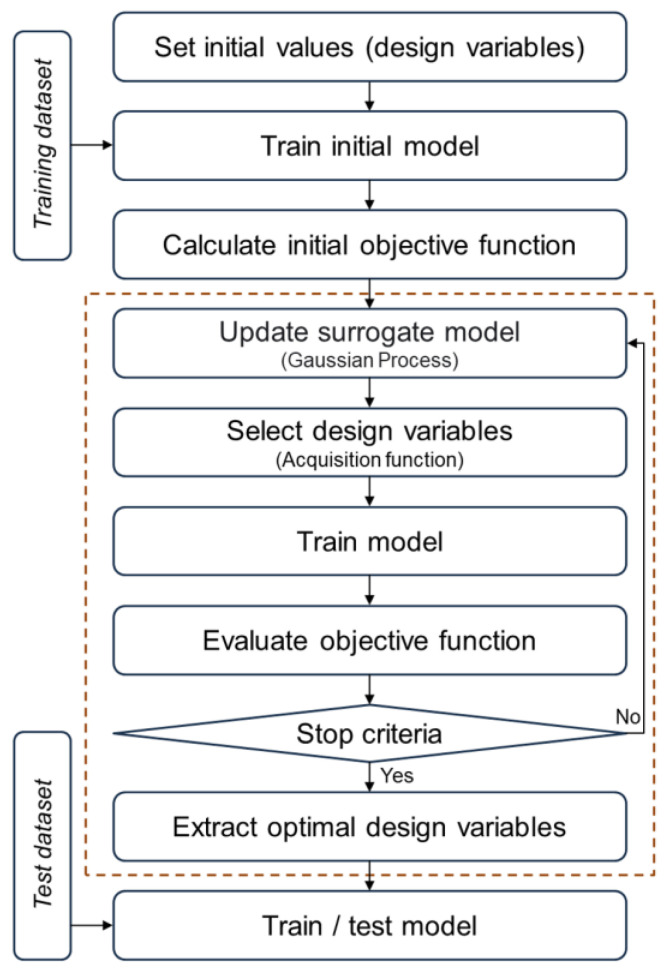
The optimization process.

Figure 4 illustrates the optimization approach that this study proposes. The dataset is utilized to train each model. For the model detecting L3 slices, the dataset comprises CT images and their respective class labels that indicate the presence or absence of an L3 slice. For the algorithm segmenting body compositions in abdominal CT scans, the dataset is composed of CT data paired with indexed labels for each CT image. The design variables are structured as per Equation (1):

Design variables: ***d*** = {***d****_h_*, ***d****_a_*, ***d****_c_*} (1)

where ***d****_h_* represents the hyperparameter design variables. As additional CDVs, ***d****_a_* and ***d****_c_* denote the augmentation ratio and class weight design variables, respectively. Table 1 presents the variables, types, and ranges used as hyperparameter design variables. The augmentation ratio design variable is only applied in the L3 detection algorithm. The class weight design variable corresponds to the weight values assigned to each class in the final fully connected layer of each model [35]. The ranges for the augmentation ratio and class weight design variables were based on the proportions of each class in the training dataset.

Equation (2) defines the objective function for the optimization process as the inverse of the f-1 score. Equations (3) and (4) provide the Precision and Recall, respectively, used in the computation of the f-1 score.

Objective function: 1/(f-1 score) = (Precision + Recall)/(2 × Precision × Recall)(2)

Precision = TP/(TP + FP)(3)

Recall = TP/(TP + FN)(4)

where True Positive (TP) represents, for the L3 slice detection model, correctly identified L3 data, or for the abdominal segmentation model, correctly classified CT pixels. False Positive (FP) refers to instances where non-L3 data or other CT pixels are misclassified as the correct pixel. Finally, False Negative (FN) denotes cases where target pixel classes (e.g., pixels belonging to the L3 class) are misclassified as other (non-L3) classes.

To derive the optimal design variables, a Bayesian optimization process was applied. Bayesian optimization efficiently identifies optimal design variables by utilizing a surrogate model, which is a probabilistic approximation, rather than directly evaluating the objective function [36]. This study employed a Gaussian Process to estimate the objective function values and used the acquisition function (expected improvement) to determine the next exploration point (Figure 4). This approach is particularly suited to optimization problems with high computational costs, such as the tuning of hyperparameters of complex machine learning models, as it enables the discovery of optimal values with minimal exploration [37]. After completing the Bayesian optimization process, the optimal design variables were extracted, then used to train the model.

## 3. Dataset

**Figure 5:**
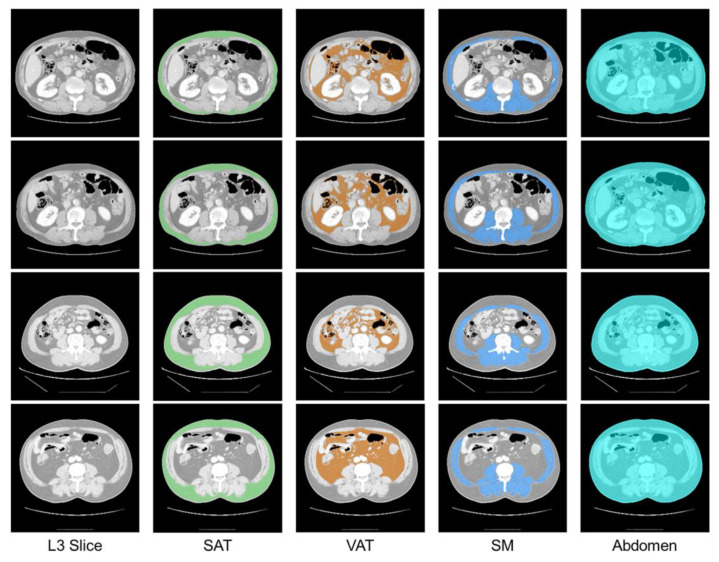
Samples of CT scans with manually segmented abdominal tissues. From left to right, each column presents the original L3 slice, segmented subcutaneous adipose tissue (SAT, light green), visceral adipose tissue (VAT, orange), skeletal muscle (SM, blue), and the combined abdominal segmentation (cyan). Each tissue type is color-coded to enhance visual distinction.

This study was a retrospective analysis utilizing CT data privately collected from 150 prostate or bladder cancer patients at the Department of Urology of Gangneung Asan Hospital, without restrictions on data selection. Patients were included if medical information such as age, weight, and underlying diseases, and prostate cancer or bladder cancer were available. Table 2 presents the clinical characteristics of patients. The median age of the patients was 67.5 years (IQR: 62.3–73.0) and median BMI was 24.5 kg/m^2^ (IQR: 22.6–26.2). The Institutional Review Board of Gangneung Asan Hospital approved the research protocol, which adhered to the principles of the Declaration of Helsinki (IRB No. 2022-04-013). The CT scans were acquired using LightSpeed 16-slice CT scanner (GE Healthcare, Milwaukee, WI, USA) with the following parameters: 5 mm slice thickness, 120 kVp tube voltage, 250 mAs tube current, and images reconstructed using Adaptive Statistical Iterative Reconstruction and Filtered Back Projection techniques. Under the guidance of an abdominal imaging radiology specialist, L3 slices were extracted from the abdomen and pelvic CT scans of each patient. The abdomen, SM, SAT, and VAT within the extracted L3 slices were manually segmented. The software ITK-SNAP v3.8 (Free Software Foundation, 2007) was used to extract the L3 slices and segment the tissues (Figure 5).

## 4. Model Implementation

### 4.1. Preprocessing

The CT images were stored in DICOM format with dimensions of (512 × 512) pxl. The DICOM images were converted to grayscale PNG images and all pixel values were normalized to fall within the range [0, 1]. To detect the L3 slices, the Hounsfield Unit (HU) values of the CT scans were adjusted to the range [−190, 150] to enhance the visibility of muscles, fat, and bones [38,39]. By applying histogram stretching, the intensity values were then normalized by redistributing them within a specific range, to ensure better concentration of pixel intensities for further processing.

### 4.2. Implementation of L3 Slice Detection Model with Optimization

To distinguish between L3 and non-L3 slices, the L3 detection model was trained as a binary classification model. The data from 150 prostate and bladder cancer patients were randomly split 5-fold, with 30 patients per fold (comprising 2746 L3 slices and 96 non-L3 slices per fold), and designated as test data. The data from the remaining 120 patients (comprising 10,984 L3 slices and 380 non-L3 slices) were used for model training. To address performance degradation caused by class imbalance in the data, augmentation ratio and class weights as CDVs were introduced to optimize the process. To augment data, the augmentation ratio was adjusted to achieve a balanced dataset where the ratio of L3 to non-L3 slices was constrained to 1:1. Class weights were determined based on prior research, using the inverse of the square root of each class frequency as the minimum constraint [35,40,41]. This adjustment ensured that the model accounted for the imbalance in class proportions during training, mitigating bias towards the majority class.

### 4.3. Implementation of Abdominal Segmentation Model with Optimization

The deep learning model for segmenting SM, SAT, and VAT was trained to perform multi-class classification on abdominal CT slices, with classes assigned as follows: background = 0, SM = 1, SAT = 2, VAT = 3, and other regions = 4. The only input was L3 slices of the spine. To develop a generalized model, the entire dataset was divided five-fold for cross-validation. The training phase utilized data from 120 patients (380 L3 slices and their corresponding labels), while the testing phase utilized data from 30 patients (96 L3 slices and their corresponding labels). To prevent overfitting, data augmentation techniques, such as rotation, horizontal flipping, and vertical flipping, were employed. The dataset was augmented to be four times the original size. To address class imbalance, the minimum value for each class weight was set as the ratio of the number of pixels in that class to the total number of pixels in the dataset. The images were resized to (256 × 256) pxl for input into the U-Net model and normalized so that all pixel values fell within the range [0, 1]. Post-processing was applied to enhance performance by refining the predicted class of each pixel based on HU ranges, as determined by prior research. The post-processing HU criteria for SM, SAT, and VAT were [29, 150], [−150, −50], and [−190, −30], respectively [21].

The training and optimization processes were conducted using a system equipped with an RTX 2080Ti GPU, featuring 4352 CUDA cores, a base clock speed of 1665 MHz, and 11 GB of RAM. All implementations, including model training and optimization, were performed using MATLAB R2023a (MathWorks, Inc., Natick, MA, USA).

## 5. Performance Evaluation

This study evaluated the model performance using five-fold leave-one-out cross-validation. For the model detecting L3 slices, performance was assessed based on slice error. The results from a patient’s CT data were reviewed and the CT slice with the highest probability of being L3 (based on the output of the softmax layer for the L3 or non-L3 class probabilities) was selected as the predicted L3 slice. The reported result was the difference in slice position between the predicted L3 and the actual L3.

For the model classifying tissues in abdominal CT scans, the Jaccard score, Dice score, Sensitivity, Specificity, and the area values of each tissue were used to evaluate the performance [23]. Equation (5) shows the Jaccard score that divides the size of their intersection by the size of their union to measure the similarity between two sets, while Equation (6) shows the Dice coefficient that quantifies the overlap between the predicted segmentation and the ground truth segmentation to provide a measure of their agreement; the Dice coefficient range is (0–1), where 0 indicates no overlap and 1 indicates perfect agreement. The Mean Surface Distance (MSD) was used to evaluate segmentation accuracy (Equation (7)). MSD calculates the average Euclidean distance between the surfaces of the predicted segmentation and the ground truth, providing a boundary-sensitive metric. In this context, ∂A and ∂B represent the boundaries of the predicted and ground truth segmentations, respectively, and ∥x − y∥ denotes the Euclidean distance between corresponding surface points.

Jaccard score = TP/(FN + TP + FN)(5)

Dice score = (2 × TP)/(2 × TP + FP + FN)(6)

(7) $$\mathrm{MSD}(A,\;B)=(1/|\partial A|)\;\sum_{x\in \partial A}{min}_{\left(y\in \partial B\right)}\|x.-y\|$$

The estimated area for each body composition component (abdomen, SAT, VAT, and SM) was calculated and correlation analysis was performed between the estimated and actual measurements. The tissue areas were computed by multiplying the pixel count for each class in an abdominal CT image by the square of the physical length represented by a single pixel. To adjust for the actual physical area, the computed areas were multiplied by a factor of four, since the original image size of (512 × 512) pxl was resized to (256 × 256) pxl.

## 6. Results

**Figure 6:**
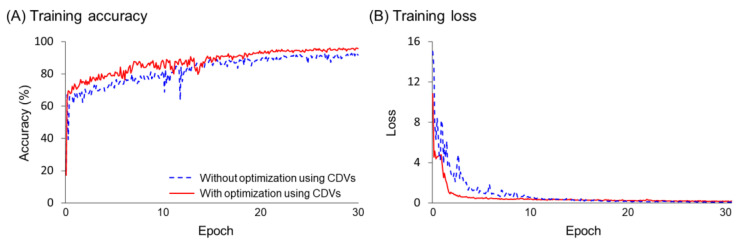
Accuracy and loss plot while training models with and without optimization using CDVs.

Figure 6 illustrates the representative trends in accuracy and loss over epochs during the training process of the models. The training accuracy of both models, with and without optimization using CDVs, stabilized in the epoch range (20–30). To prevent overfitting, training was stopped at 30 epochs. The model with optimization using CDVs converged to approximately 90% accuracy, which was about 3% higher accuracy than that of the model without optimization (Figure 6A). For training loss, the model with optimization converged rapidly within 2 to 3 epochs, while the model without optimization converged within approximately 10 epochs (Figure 6B).

**Figure 7:**
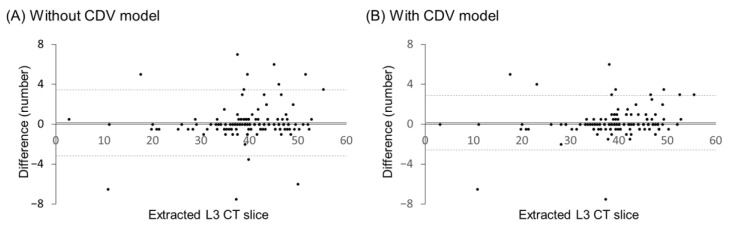
Bland–Altman plots of the extracted L3 slice number.

The Bland–Altman plots in Figure 7 compare the L3 slice detection results with and without CDV optimization. The mean differences were (0.13 and 0.15) for models without and with optimization, respectively. The standard deviation of the limits of agreement for the model without optimization was upper limit 3.42 and lower limit −3.16, whereas for the model with optimization, it was upper limit 2.87 and lower limit −2.56. The proportion of test data within the 95% consistency limits was 92% without optimization and 92.7% with optimization.

**Figure 8:**
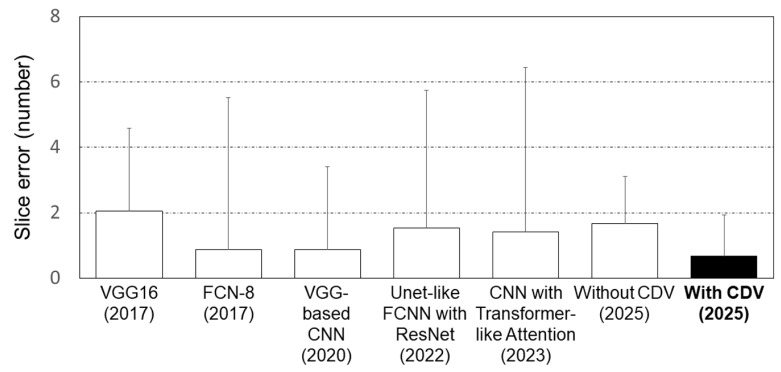
Comparison of performance between L3 slice detection models.

Figure 8 compares the performance of the L3 slice detection model in this study with that of previous studies. Previous studies reported an average error range of (0.87 to 2.05). Of these previous models, the VGG11 architecture-based transfer learning model demonstrated the best performance in L3 extraction, achieving (0.87 ± 2.54) error (Dabiri et al., 2020) [23]. In this study, the model without CDVs achieved (1.68 ± 1.43) error, while the model with CDVs showed improved performance with (0.68 ± 1.26) error, outperforming the previous studies.

**Figure 9:**
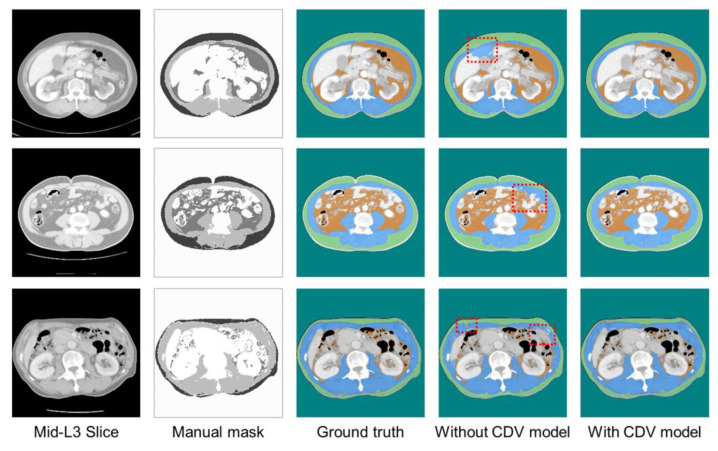
Visualization of qualitative performance of standard Unet architecture.

**Figure 10:**
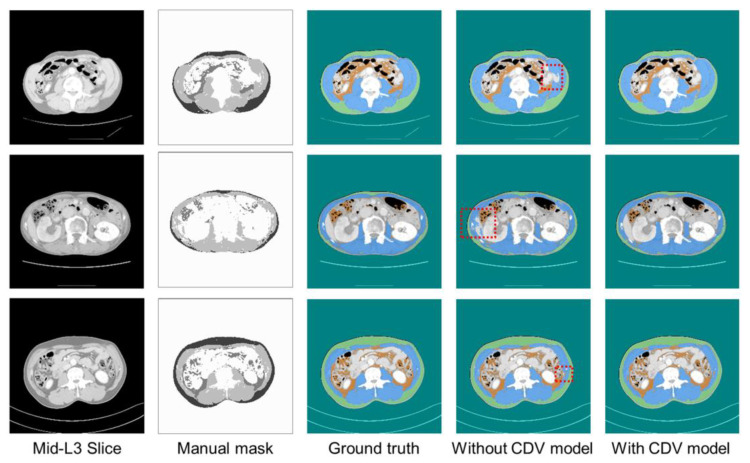
Visualization of qualitative performance of Swin-Unet architecture.

**Figure 11:**
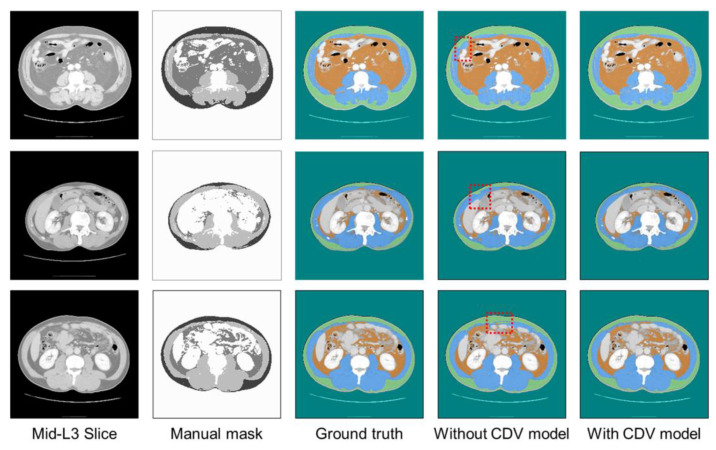
Visualization of qualitative performance of SegFormer architecture.

Figure 9, Figure 10 and Figure 11 show representative predicted segmentation maps for SM, VAT, SAT, and abdomen from the standard Unet, Swin-Unet, and SegFormer models, respectively. The same test data were visualized to compare the performance of the models with and without CDVs. The model without CDVs exhibited segmentation errors, particularly in the muscle and visceral fat regions (highlighted by red dashed boxes in the fourth column), while the model with CDVs showed segmentation that closely resembled the ground truth, demonstrating clear performance improvements. Similar segmentation results were observed across all three architectures (standard Unet, Swin-Unet, and SegFormer models).

Table 3 provides a comprehensive performance comparison of three segmentation models—standard Unet, Swin-Unet, and SegFormer—with and without the application of CDVs. Across all models and tissue types, the incorporation of CDVs consistently improved performance metrics such as Jaccard score, Dice coefficient, sensitivity, specificity, and MSD. Notably, the SegFormer model with CDVs achieved the highest accuracy, with a Dice coefficient of 0.987 and the lowest MSD of 0.279 for abdominal tissue segmentation. Among the tissue types, SAT and SM showed significant improvement when CDVs were applied, particularly in reducing boundary discrepancies as indicated by lower MSD values. Similar trends were observed for VAT, SAT, and abdominal tissue, suggesting that the CDVs enhance performance across various body composition segmentations.

**Figure 12:**
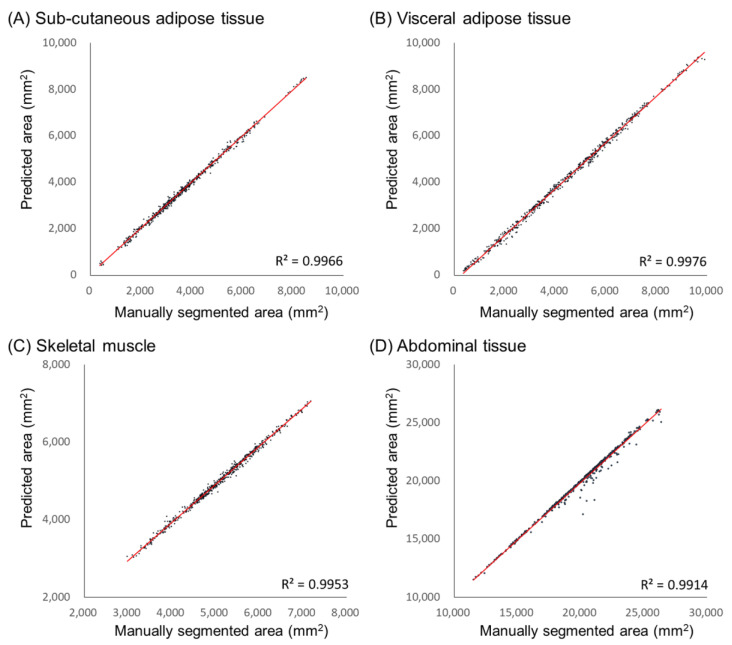
Scatter plot between manual segmented and model predicted tissue areas.

Figure 12 presents scatter plots of the predicted vs. measured areas for each tissue type in abdominal CT scans. The measured area ranges for SAT, VAT, SM, and abdomen were ((370–8400), (360–9900), (2900–7200), and (11,500–26,200)) mm^2^, respectively. Correlation analysis between the clinician-measured and model-predicted tissue areas showed high coefficients of determination (R^2^), with values for SAT, VAT, SM, and abdomen of (0.9966, 0.9976, 0.9953, and 0.9914), respectively.

## 7. Discussion

Muscle and fat measurement data obtained from body composition analysis are emerging as promising new biomarkers for early cancer diagnosis and prognosis [13]. Traditionally, body composition analysis has been used to assess the proportions of muscle and fat, evaluate nutritional status, and manage obesity-related issues, such as metabolic diseases and hypertension [42]. Recent studies suggest that changes in body composition, such as sarcopenia—a condition characterized by fatty infiltration into muscles and a reduction in skeletal muscle mass—can increase the risk of cancer, worsen cancer prognosis, and amplify the toxicity of chemotherapy [43,44,45]. These findings underscore the importance of accurate measurement of muscle and fat mass through body composition analysis as essential biomarkers for early cancer diagnosis and treatment [45,46,47,48,49].

This study proposes an optimization process for training deep learning-based models for L3 slice detection and the automatic segmentation of abdominal CT data to evaluate body composition. Early detection and the rehabilitation of cancer have been the subjects of intense research and sarcopenia diagnosis is known to help predict prognosis [18]. While body composition analysis for diagnosing sarcopenia is actively being studied, it has yet to be widely adopted in clinical practice [50]. This is likely due to the high cost and time required to segment body composition and skeletal muscle mass. However, body composition analysis using deep learning models holds significant potential for future clinical applications. Thus, fully automating the segmentation of muscle and fat within L3 slices could offer a valuable tool to assess cancer risk [18,43,45].

This study developed a deep learning model to detect L3 slices, achieving slice errors of (1.68 ± 1.43) without CDVs and (0.68 ± 1.26) with CDVs. Considering that radiologists typically annotate within a one-slice margin of error, the performance of the model with CDV is adequately precise. Further, it demonstrates comparable performance to existing studies on L3 slice detection, which report slice errors ranging (0.87 to 2.05). Dabiri et al. (2020) addressed class imbalance between non-L3 and L3-level datasets by training models with additional non-L3-level data from other datasets [23]. However, when additional datasets are unavailable, this approach is challenging. Belharbi et al. (2017) proposed an algorithm using maximum intensity projection to compress entire CT scans into a single image, then employing a sliding window to input segmented images into a regression model for L3 slice detection [19]. However, when detecting L3 slices in patients with spinal curvature or deformities, that model faces limitations [51]. In contrast, by augmenting L3 slices and adjusting class weights in the loss function without requiring additional data, our model with CDV addresses these limitations, achieving similar performance to prior studies.

The abdominal segmentation algorithm developed for body composition estimation achieved a mean Dice coefficient of (0.971 ± 0.001). Previous studies have reported high accuracy in L3-level automatic tissue segmentation, with most models achieving results comparable to those of expert annotators (Dice score = 0.9) [18,52,53,54]. Although differences in datasets make direct model-to-model comparisons difficult, the performance of our L3 segmentation algorithm exceeds a Dice score of 0.9, aligning with prior findings. Compared to traditional methods, deep learning-based algorithms also offer a significant reduction in processing time, taking approximately 3 min for abdominal CT segmentation, which makes them an efficient alternative for clinical practice [21].

The proposed CDVs incorporate two methods to address performance degradation caused by class imbalance within datasets: re-sampling and cost-sensitive re-weighting. The resampling method involves oversampling to increase the number of minor class samples. Both methods vary in their optimal variables according to the dataset and deep learning model used. In this study, the augmentation ratios for each dataset and class weights within the loss function were treated as design variables and a performance-driven optimization algorithm was constructed. For the L3 slice detection model, the augmentation ratio for the L3 class range was (17 to 24) %, relative to the non-L3 class. However, cost-sensitive re-weighting showed inconsistent results for both L3 slice detection and segmentation models. This aligns with the general finding that in AI models, no universally optimal values exist for data augmentation and cost-sensitive re-weighting [24,55]. Nonetheless, our study demonstrates that systematically optimizing these hyperparameters through CDVs can improve model performance while maintaining computational efficiency.

The clinical significance of this improvement lies in the automation of accurate and reliable body composition analysis, which plays a crucial role in cancer prognosis pre-diction, chemotherapy toxicity assessment, and nutritional evaluation in oncology patients. By improving segmentation accuracy while maintaining computational efficiency, our method reduces the need for manual corrections by radiologists, thereby saving time and minimizing inter-operator variability. Additionally, accurate segmentation of muscle and fat distribution within the L3 slice has been linked to patient survival outcomes, making the precision of this task highly relevant to clinical decision-making [45,46,47,48]. Unlike previous methods that rely on either manual segmentation or less optimized deep learning models, our approach enhances the reliability of muscle and fat quantification, potentially contributing to better treatment planning and patient monitoring. Furthermore, our optimization strategy ensures greater generalizability across different datasets, which is a crucial factor for real-world clinical adoption. Since body composition analysis is increasingly being integrated into oncological assessments, the ability to automate and standardize this process using deep learning can facilitate its broader clinical application. These findings suggest that our proposed optimization techniques could be applied to other medical image segmentation tasks where class imbalance poses a challenge, further improving the efficiency and accuracy of AI-driven diagnostics.

This study has limitations, as follows. First, specific deep learning backbones such as ResNet, Unet, and SegFormer architecture were used for the L3 slice detection and segmentation tasks. It is important to validate the proposed optimization-integrated deep learning strategy by applying it to a variety of models. Second, the dataset used in this study consisted of CT slices with a fixed spacing of 5 mm, while no datasets with varying slice intervals were included; future research should thus aim to ensure model robustness by incorporating datasets that have been acquired from various devices and with different slice intervals. Similarly, the dataset consisted of prostate and bladder cancer patients with a mean age of 67.8 years; expanding the training dataset to include data from healthy individuals and younger populations with diverse muscle and fat distributions in L3 slices could further enhance the applicability of the model.

In conclusion, this study proposes deep learning models capable of detecting the L3 slice in CT images and automatically segmenting body composition. In particular, the issue of class imbalance in the deep learning models was addressed by applying an optimization process that adjusts oversampling and class weights as CDVs, preventing performance degradation. The CT images used in this study were from a total of 150 prostate cancer and bladder cancer patients. A ResNet50 classifier detects the L3 slice, while standard Unet, Swin-Unet, and SegFormer models segment abdominal tissues. As a result, the detection error for the L3 slice was approximately 0.68 ± 1.26 and the body composition segmentation showed a Dice score averaging 0.987 ± 0.001, demonstrating improved performance compared to the models that did not incorporate correction design variables. This study indicates that the body composition in the L3 region can be automatically segmented with improved performance. It suggests that this method may be used as a biomarker for early cancer diagnosis and treatment in clinical practice.

## Figures and Tables

**Table 1:** Hyperparameter type and range.

Parameters	Type	Range
L2Regularization	Logarithmic (continuous)	[0.0001, 0.01]
InitialLearningRate	Logarithmic (continuous)	[0.0001, 0.01]
Batchsize	Integer (discrete)	[10, 32]
GradientThreshold	Integer (discrete)	[1, 6]
Epoch	Integer (discrete)	[5, 20]
Momentum	Real (continuous)	[0.7, 0.99]

**Table 2:** Clinical characteristics of patient cohort.

		All Patients (n = 150)
Disease	Prostate cancer, n (%) Bladder cancer, n (%)	104 (69.3) 46 (30.7)
Sex	Male, n (%), female, n (%)	142 (94.7), 8 (5.3)
Age	Median age, yr (IQR)	67.5 (62.2–73.0)
BMI	Median BMI, kg/m^2^ (IQR)	24.5 (22.6–26.2)
Height	Median height, cm (IQR)	165.0 (161.1–168.7)
Weight	Median weight, kg (IQR)	66.4 (60.1–72.3)
DM	n (%)	37 (24.7)
HTN	n (%)	67 (44.7)

IQR, interquartile range; BMI, body mass index; DM, diabetes mellitus; HTN, hypertension.

**Table 3:** Quantitative performance metrics between the trained models (standard Unet, Swin-Unet, and SegFormer architectures) without and with CDVs.

		Tissue	Jaccard Score	Dice Coefficient	Sensitivity	Specificity	MSD
Standard Unet	Without CDVs	SM	0.908 ± 0.006	0.950 ± 0.004	0.944 ± 0.003	0.994 ± 0.001	1.027 ± 0.210
		VAT	0.871 ± 0.005	0.906 ± 0.004	0.936 ± 0.011	0.989 ± 0.002	1.721 ± 0.261
		SAT	0.912 ± 0.004	0.945 ± 0.003	0.965 ± 0.009	0.995 ± 0.001	0.812 ± 0.188
		Abdomen	0.963 ± 0.001	0.981 ± 0.001	0.976 ± 0.004	0.957 ± 0.006	0.310 ± 0.121
	With CDVs	SM	0.945 ± 0.001	0.971 ± 0.001	0.981 ± 0.003	0.996 ± 0.001	0.618 ± 0.234
		VAT	0.898 ± 0.003	0.924 ± 0.002	0.963 ± 0.007	0.996 ± 0.001	1.287 ± 0.168
		SAT	0.960 ± 0.001	0.976 ± 0.001	0.980 ± 0.004	0.998 ± 0.001	0.354 ± 0.153
		Abdomen	0.974 ± 0.001	0.987 ± 0.001	0.988 ± 0.001	0.976 ± 0.002	0.312 ± 0.134
Swin-Unet	Without CDVs	SM	0.936 ± 0.005	0.967 ± 0.003	0.969 ± 0.003	0.992 ± 0.001	0.754 ± 0.093
		VAT	0.875 ± 0.012	0.933 ± 0.007	0.922 ± 0.006	0.994 ± 0.001	0.692 ± 0.188
		SAT	0.899 ± 0.008	0.947 ± 0.004	0.953 ± 0.006	0.994 ± 0.001	0.582 ± 0.177
		Abdomen	0.964 ± 0.002	0.981 ± 0.001	0.982 ± 0.001	0.951 ± 0.004	0.375 ± 0.133
	With CDVs	SM	0.952 ± 0.004	0.975 ± 0.002	0.981 ± 0.003	0.977 ± 0.001	0.588 ± 0.080
		VAT	0.903 ± 0.012	0.949 ± 0.006	0.973 ± 0.002	0.995 ± 0.001	0.442 ± 0.145
		SAT	0.962 ± 0.005	0.975 ± 0.003	0.974 ± 0.003	0.992 ± 0.001	0.404 ± 0.126
		Abdomen	0.982 ± 0.001	0.986 ± 0.001	0.981 ± 0.001	0.997 ± 0.001	0.279 ± 0.113
SegFormer	Without CDVs	SM	0.942 ± 0.007	0.970 ± 0.004	0.984 ± 0.001	0.996 ± 0.001	0.325 ± 0.115
		VAT	0.821 ± 0.018	0.901 ± 0.011	0.930 ± 0.008	0.986 ± 0.001	0.847 ± 0.171
		SAT	0.914 ± 0.006	0.955 ± 0.003	0.975 ± 0.002	0.992 ± 0.001	0.585 ± 0.138
		Abdomen	0.960 ± 0.002	0.979 ± 0.001	0.971 ± 0.001	0.970 ± 0.003	0.385 ± 0.143
	With CDVs	SM	0.957 ± 0.002	0.978 ± 0.001	0.989 ± 0.003	0.987 ± 0.001	0.548 ± 0.193
		VAT	0.907 ± 0.008	0.951 ± 0.002	0.975 ± 0.001	0.992 ± 0.001	0.967 ± 0.316
		SAT	0.969 ± 0.002	0.975 ± 0.001	0.975 ± 0.001	0.985 ± 0.001	0.416 ± 0.228
		Abdomen	0.986 ± 0.001	0.987 ± 0.001	0.992 ± 0.001	0.994 ± 0.001	0.376 ± 0.141

## Data Availability

The data used in this study are not available for public sharing due to privacy and confidentiality considerations. However, if there are reasonable requests, the code and data can be made available with the authors’ consent.
